# Acute Liver Failure Secondary to Pyogenic Hepatic Abscess

**DOI:** 10.7759/cureus.34258

**Published:** 2023-01-27

**Authors:** Emad Elmusa, Muhammad Waleed Raza, Michael Orlando, Seth Boyd, Robert Kulchinsky

**Affiliations:** 1 Internal Medicine, HCA Florida (FL) Orange Park Hospital, Orange Park, USA; 2 Transitional Year (TY), HCA Florida (FL) Orange Park Hospital, Orange Park, USA

**Keywords:** fulminant hepatitis, acute fulminant hepatitis, acute fulminant liver failure, bacterial liver abscess, liver abscess drainage, acute liver failure (alf), pyogenic hepatic abscess, hepatic abscess

## Abstract

Hepatic abscesses are rare and can be pyogenic or amebic. Pyogenic hepatic abscesses are treated with antibiotics, percutaneous drainage when larger than 5 cm, and rarely requires surgical treatment. Clinical and laboratory manifestations of pyogenic hepatic abscesses include fever, abdominal pain, and elevations in liver enzymes. There is little documentation that a pyogenic hepatic abscess can cause acute liver failure. We present a case of a patient who developed acute liver failure secondary to a 14 cm pyogenic liver abscess. The patient’s hepatic function normalized with percutaneous drain placement and antibiotics.

## Introduction

The incidence of hepatic abscesses from 1973 to 1993 in the United States was estimated to be 20/100,000 [1]. The majority of hepatic abscesses are bacterial in origin and are most commonly secondary to lithiasic biliary pathology [1]. Diagnosis of hepatic abscesses relies on imaging [1]. Typical manifestations of pyogenic hepatic abscesses include fever and abdominal pain [1]. Upon literature review, there is scarce documentation of a pyogenic hepatic abscess causing acute liver failure. We present a case of a patient with a 14 cm pyogenic liver abscess who developed acute liver failure. The patient’s acute liver failure resolved after drain placement and antibiotics.

## Case presentation

An 87-year-old male with a past medical history significant for hypertension, coronary artery disease status post coronary artery bypass grafting, atrial fibrillation on apixaban, high degree atrioventricular block status post permanent pacemaker, benign prostatic hyperplasia, and a history of melanoma status post resection one year prior, presented with a chief complaint of shortness of breath. The patient denied recent travel, fevers, abdominal pain, nausea, vomiting, diarrhea, and weight loss.

**Figure 1:**
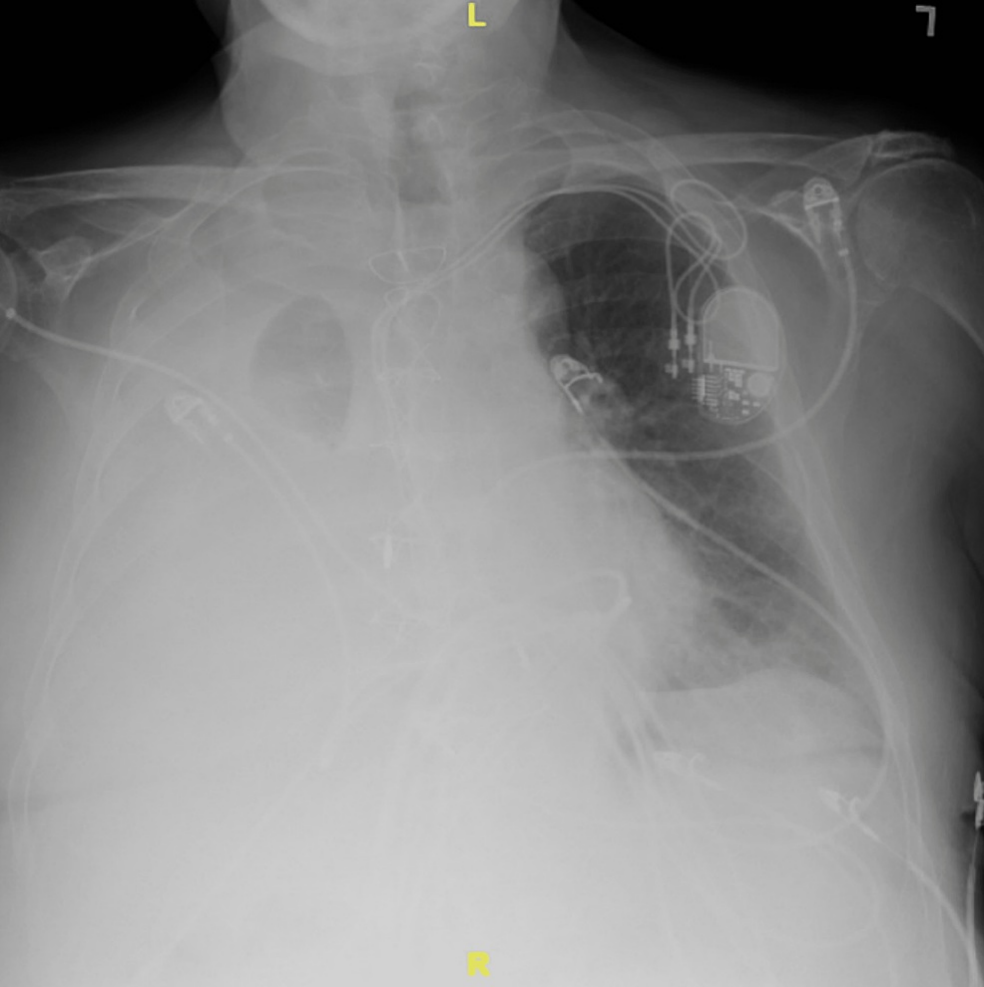
Chest radiograph with large right pleural effusion.

**Figure 2:**
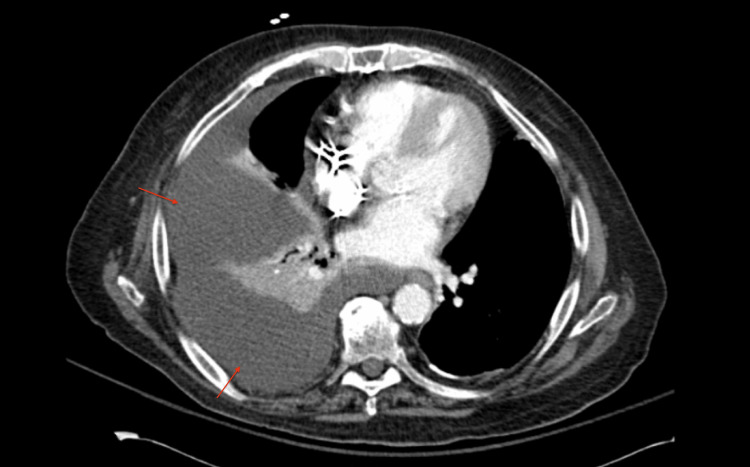
Axial CT with contrast showing large right pleural effusion and adjacent atelectasis.

**Figure 3:**
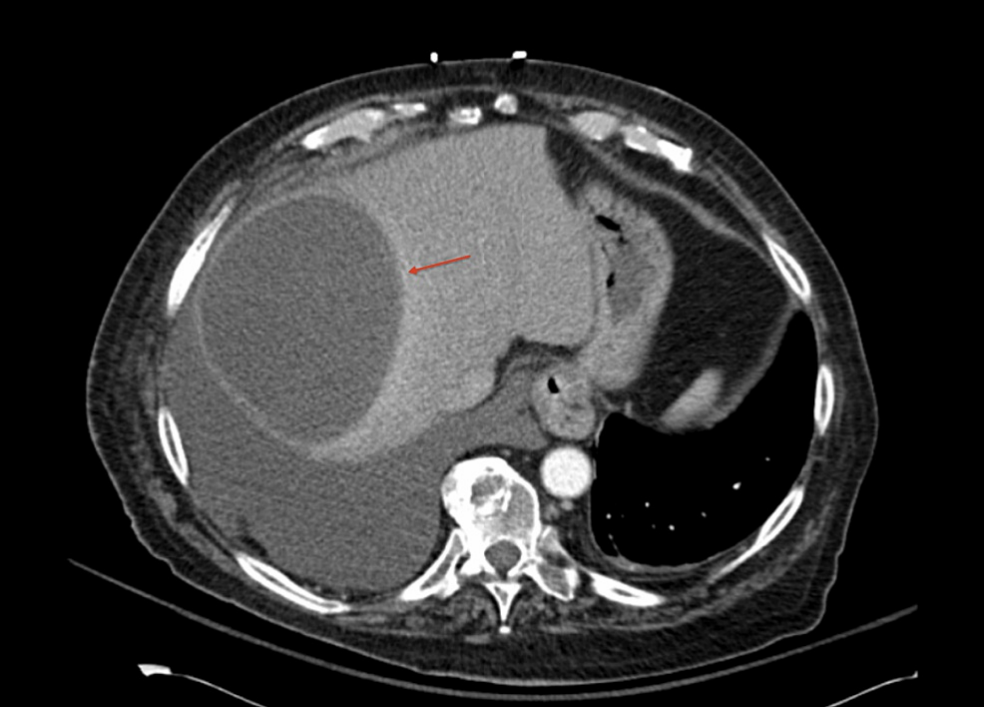
Axial CT with contrast showing 14.2 cm well-defined hypodense lesion in the right liver lobe.

**Figure 4:**
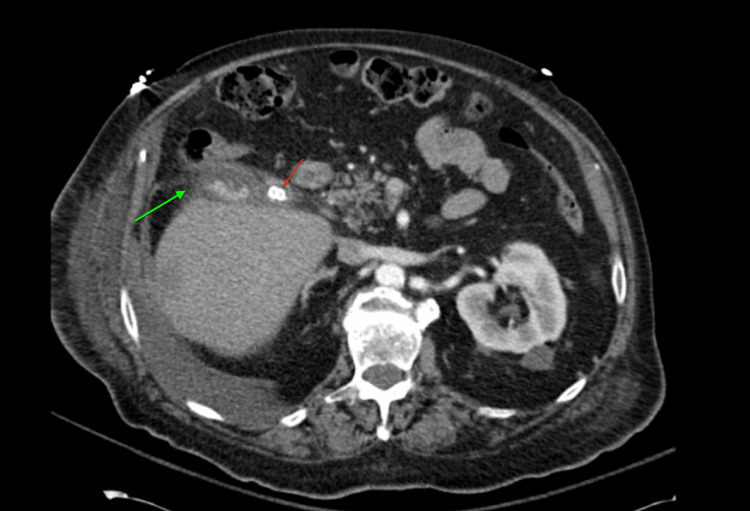
Axial CT with contrast showing pericholecystic fat stranding (green arrow) and cholelithiasis (red arrow).

On presentation in the emergency department, vitals revealed a temperature of 36.9°C, heart rate of 64 bpm, respiratory rate of 18 bpm, blood pressure of 162/64 mmHg, and pulse oxygen saturation of 96%. On physical exam, the patient’s abdomen was soft, non-tender, and non-distended. Initial laboratory values are shown in Table 1. Chest radiograph revealed a large pleural effusion on the right (Figure 1). CT of the chest with contrast demonstrated a large right pleural effusion, a 14.2 cm well-defined hypodense lesion in the right liver lobe, pericholecystitis, and cholelithiasis (Figures 2-4). 

**Table 1:** Laboratory values on admission. AST, aspartate transaminase; ALT, alanine transaminase; PT, prothrombin time; INR, international normalized ratio; WBC, white blood count

Lab test	Result	Reference range
Total bilirubin	1.2 mg/dL	0.2-1.1 mg/dL
AST	39 U/L	0-34 U/L
ALT	37 U/L	10-49 U/L
PT	17.4 s	10.2-12.9 s
INR	1.51	
Brain natriuretic peptide	177.79 pg/mL	0-125 pg/mL
WBC	12.5 x 10^3^/uL	4.0-10.5 x 10^3^/uL
C-reactive protein	28 mg/L	< 10 mg/L

It was unclear what the hepatic lesion represented. Given the patient’s white blood cell count and history of melanoma, the primary differential included infection and malignancy. The patient was started on empiric broad-spectrum antibiotics including vancomycin, cefepime, and metronidazole. 

The patient underwent right-sided thoracentesis yielding 1200 mL of clear yellow fluid. The pleural lactate dehydrogenase (LDH) measured 325 U/L and serum LDH measured 200 U/L (reference 120-246), suggesting an exudative effusion. Cytology was ultimately negative for malignancy. Pleural and blood bacterial cultures and pleural fungal and acid-fast bacilli cultures were negative. 

**Figure 5:**
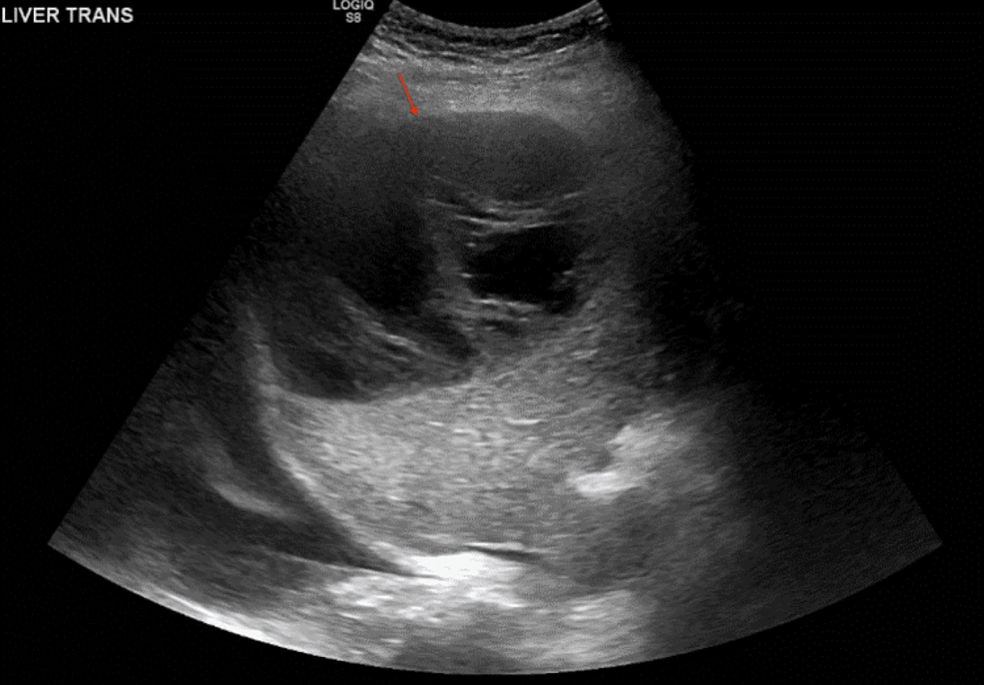
Ultrasound showing well-defined right hepatic lesion with heterogenous echogenicity with thick internal septations.

Further evaluation with ultrasound again showed a 14 cm heterogenous echogenic liver lesion with thick internal septations without any internal doppler flow (Figure 5). 

**Figure 6:**
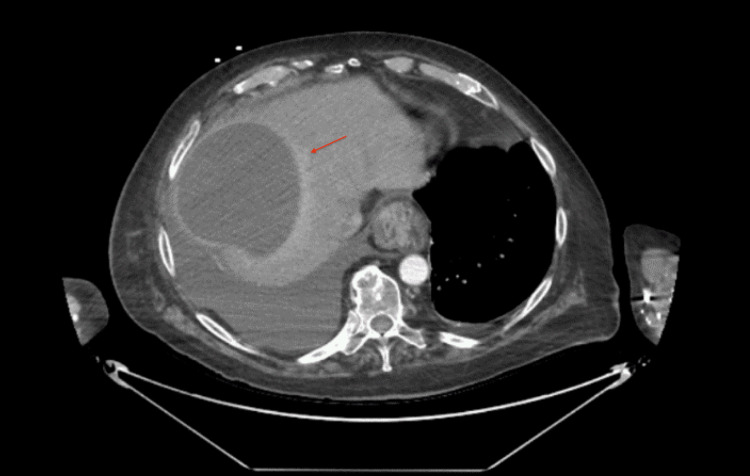
Triple-phase liver CT with hypodense right hepatic lobe lesion with ring enhancement on the late arterial phase.

In order to differentiate between malignancy and abscess, the patient underwent triple-phase liver protocol CT. On the late arterial phase, the lesion showcased ring enhancement (Figure 6), favoring abscess. Further workup revealed an alpha fetoprotein of < 2.1 ng/mL (reference 0.0-8.0 ng/mL) and a carcinoembryonic antigen (CEA) of 12.1 ng/mL (reference 0.0-5.0 ng/mL). The CEA was mildly elevated and outpatient colonoscopy was recommended. 

**Figure 7:**
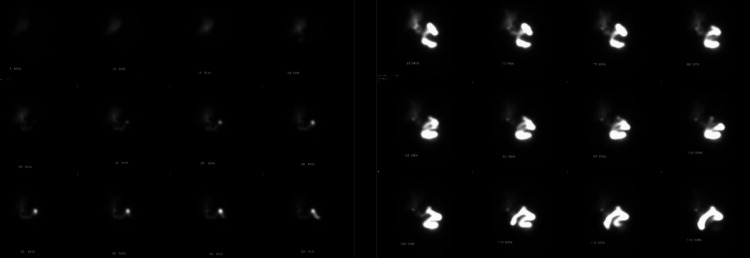
Normal HIDA scan. HIDA, hepatobiliary iminodiacetic acid

The patient’s primary risk factor for developing a hepatic abscess was cholelithiasis and radiologic evidence suggesting cholecystitis. However, the patient denied abdominal pain, nausea, and vomiting. General surgery evaluated the patient and recommended a hepatobiliary iminodiacetic acid (HIDA) scan. HIDA scan revealed gallbladder activity and no bile duct obstruction (Figure 7). Therefore, it was suspected that the patient previously had an obstructing gallstone which passed.

On hospital day 8, percutaneous aspiration and drainage of the hepatic lesion was planned. However, the patient’s prothrombin time (PT), international normalized ratio (INR), aspartate transaminase (AST), and alanine transaminase (ALT) values were worsening (Table 2). The patient’s home apixaban was still being given, however, coagulation studies on admission were markedly lower. Additionally, the patient exhibited grade 1 hepatic encephalopathy, evidenced by insomnia, anorexia, and malaise. The patient denied any previous liver disease. He denied prior alcohol abuse and denied any alcohol use in the past couple of years. Hepatitis serologies, including hepatitis A IgM antibody, hepatitis B surface antigen, hepatitis B core IgM antibody, and hepatitis C antibody were negative. These findings in conjunction were diagnostic of acute liver failure. The patient was given 10 mg of oral vitamin K. The patient’s PT and INR down trended to 22.8 s and 1.95 the following day. The patient was maintained on apixaban.

**Table 2:** Laboratory values on hospital day 8. PT, prothrombin time; INR, international normalized ratio; AST, aspartate transaminase; ALT, alanine transaminase

Lab test	Result on admission	Result on hospital day 8	Reference range
PT	17.4 s	32.8 s	10.2-12.9 s
INR	1.51	2.78	
AST	39 U/L	91 U/L	0-34 U/L
ALT	37 U/L	100 U/L	10-49 U/L

**Figure 8:**
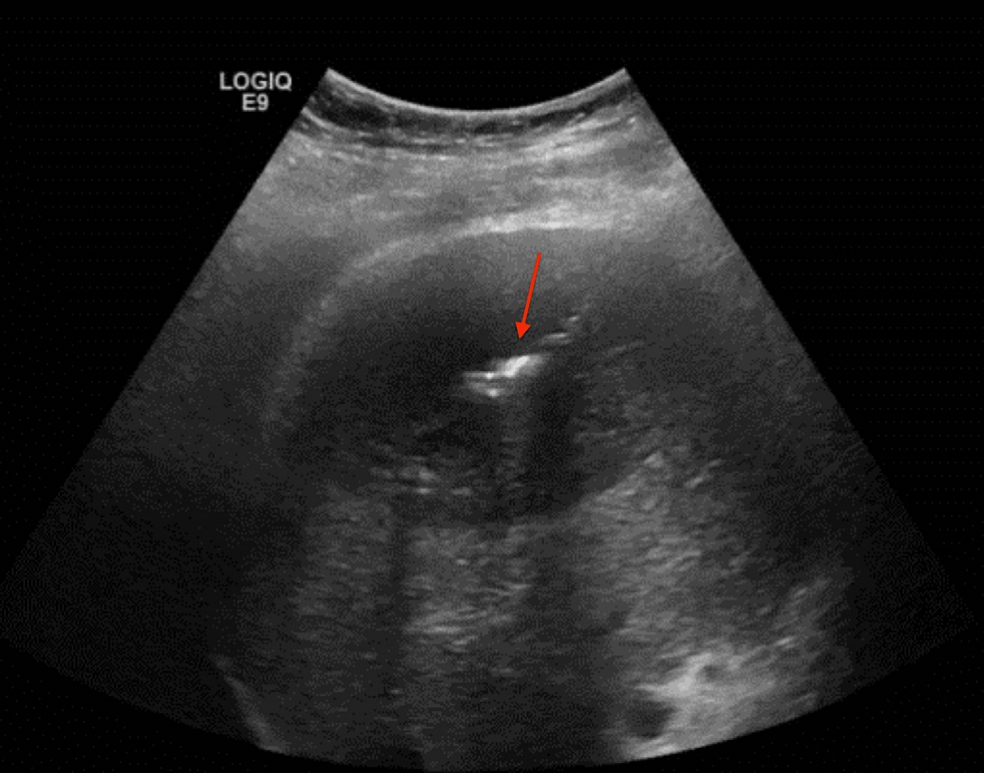
Ultrasound-guided needle aspiration and drain placement of hepatic abscess.

**Figure 9:**
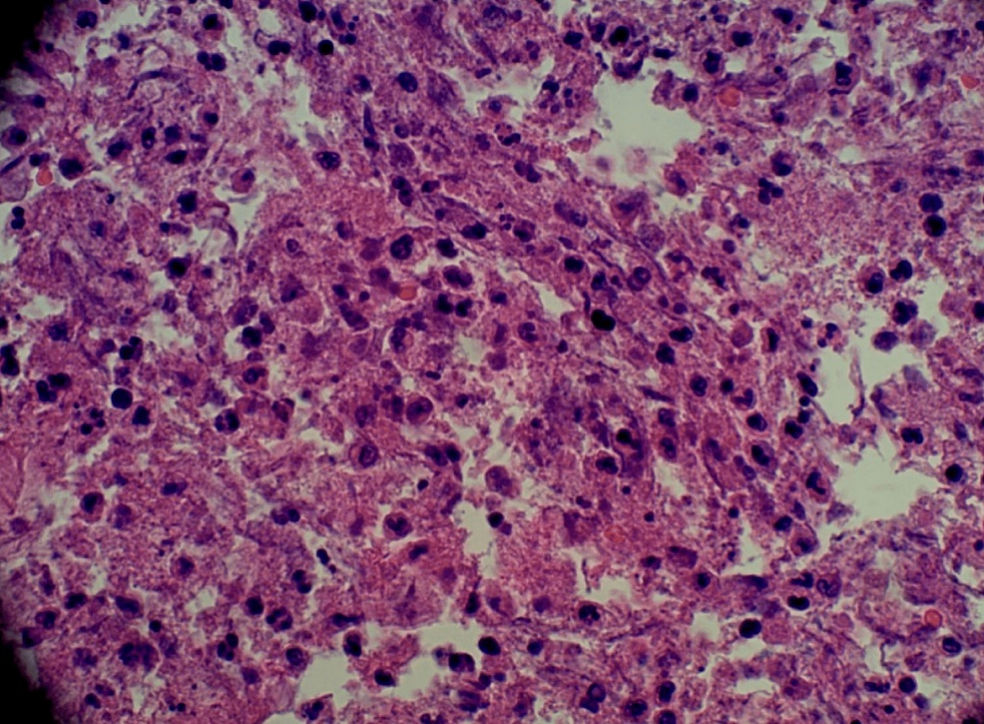
Liver fluid aspirate - H&E stain 60x magnification showing inflammatory cells (neutrophils, lymphocytes, macrophages, and debris), diagnostic of pyogenic abscess.

Interventional radiology successfully placed a hepatic abscess drain via ultrasound guidance on hospital day 9 (Figure 8). The liver fluid aspirate histopathology was consistent with a pyogenic abscess (Figure 9). Grossly, the fluid appeared purulent. Culture data was negative. However, the patient had been on empiric antibiotics since admission.

**Figure 10:**
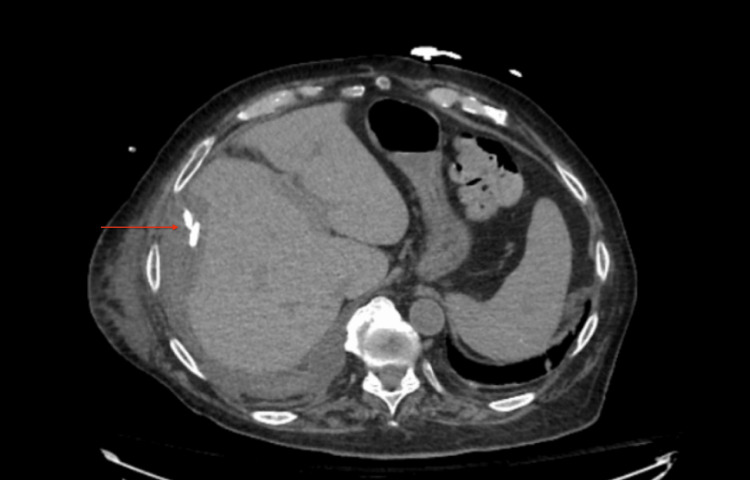
Markedly reduced size of pyogenic hepatic abscess with drain in place.

The patient’s PT and INR remained around 13 s and 1.2 for the remainder of his hospitalization. The patient’s anorexia, insomnia, and malaise improved. Liver enzymes normalized. Repeat CT showed improvement in the size of the hepatic abscess (Figure 10). The patient’s drain remained in place until output was below 20 mL/24 h and empiric antibiotics remained until the drain was removed.

## Discussion

Hepatic abscesses are rare and approximately 80% are bacterial in the United States [1]. Less commonly, hepatic abscesses can be amebic, which is caused by the extraintestinal spread of Entamoeba histolytica [2]. Pyogenic hepatic abscesses are primarily related to lithiasic biliary disease [1]. Approximately 50%-60% of pyogenic hepatic abscesses are biliary in origin [1]. Other less common etiologies include appendicitis, diverticulitis, post-surgical infections, trauma, super-infected malignancy, cryptogenic, and bacteremia [1]. Cultures can be negative in 30% of cases if antibiotics are started before drawing the abscess cultures [2]. Without treatment, mortality is around 15% [1]. Untreated, pyogenic hepatic abscesses can rupture, resulting in septic shock and death [2]. Risk factors for the development of pyogenic hepatic abscesses include diabetes, malnutrition, advanced age, and immunosuppression [2]. Additionally, pyogenic liver abscesses are more prevalent in men than women with an odds ratio of 1.85 [2]. Diagnosis is made with imaging [1]. Ultrasound and CT are the best modalities, achieving the diagnosis of a pyogenic hepatic abscess in approximately 90% of cases [1]. Ultrasound has 94% sensitivity but is unable to diagnose smaller abscesses [2]. On ultrasound, pyogenic hepatic abscesses can be hyper or hypoechoic and can have internal debris or septations [2]. CT sensitivity approaches 100% and also allows for differentiation of an abscess from a tumor [2]. Pyogenic hepatic abscesses have pre-suppurative and suppurative phases and each appears differently on CT. Pre-suppurative pyogenic hepatic abscesses appear heterogeneous, hypodense, and have irregular contours on CT, mimicking a tumor [1-2]. The suppurative phase can be multiloculated with more rounded contours and can have a peripheral enhancement or ring enhancement on CT [1]. The CT finding, regarded as quasi-pathognomonic for a hepatic abscess, is the presence of internal gas [1]. 

The most common presenting symptoms of a pyogenic hepatic abscess are fever, chills, and abdominal pain [2]. Laboratory investigation can reveal increased AST and ALT in 50% of cases [2]. Treatment depends on the size of the pyogenic hepatic abscess as well as the underlying etiology. As discussed, source control is key. Therefore, in the setting of cholecystitis or cholangitis, the gallbladder or gallstone should be addressed along with the abscess. With regard to size, if 3-5 cm, antibiotics alone are sufficient. Empiric antibiotics are selected to cover against Escherichia coli, Streptococcus species, Klebsiella pneumoniae, and Enterococcus species [2]. However, when larger than 5 cm, sonographic or CT-guided aspiration and drainage is the first-line treatment [1]. Drainage is more effective than needle aspiration, with a success rate of 100% vs 60% [1, 3]. Aspiration and drainage also give the benefit of culture data. Surgical treatment is indicated for percutaneous drainage failure, persistent sepsis, multiloculated abscesses, multiple liver abscesses, or thick purulent material not amenable to drainage [2]. 

Our patient developed acute liver failure in the setting of a large pyogenic hepatic abscess. It is most likely that mass effect alone was the primary driving force in causing acute liver failure. This is evidenced by resolution of liver function with drain placement and antibiotics. Acute liver failure can be secondary to viruses, drugs, ischemic injury, and neoplastic infiltration. However, it is rarely described to be secondary to a hepatic abscess. Acute liver failure manifests with hepatic dysfunction, abnormal liver serologies, coagulopathy, and encephalopathy [4]. There are multiple grading systems of acute liver failure including the O’Grady, Bernuau, and the Japanese system [3]. Each defines the time in weeks from jaundice to encephalopathy. Our patient would likely be categorized with hyperacute liver failure according to the O’Grady system because of minimal jaundice and features of hepatic encephalopathy. The confounder in interpreting his PT and INR is the patient’s use of apixaban. However, as shown in the retrospective observational analysis of adult patients receiving at least one dose of apixaban, the median INR ranged from 1.5 to 1.7 [5]. Our patient had an INR of 2.78 on hospital day 8. The patient’s coagulopathy, abnormal liver serologies, West Haven criteria grade 1 hepatic encephalopathy [6] in conjunction with no prior liver disease was diagnostic of acute liver failure. The patient’s acute liver failure resolved with drainage and antibiotics.

## Conclusions

Hepatic abscesses are rare and are most commonly bacterial in the United States. The majority of pyogenic hepatic abscesses are secondary to lithiasic biliary pathology. Clinical manifestations are typically fever and abdominal pain. However, as demonstrated in this case, when a pyogenic hepatic abscess is large enough, the mass effect can result in acute liver failure. However, this is reversible with drainage and antibiotics.

## References

[1] Lardière-Deguelte S, Ragot E, Amroun K (2015). Hepatic abscess: diagnosis and management. J Visc Surg.

[2] Roediger R, Lisker-Melman M (2020). Pyogenic and amebic infections of the liver. Gastroenterol Clin North Am.

[3] Cai YL, Xiong XZ, Lu J (2015). Percutaneous needle aspiration versus catheter drainage in the management of liver abscess: a systematic review and meta-analysis. HPB (Oxford).

[4] Bernal W, Wendon J (2013). Acute liver failure. N Engl J Med.

[5] Kovacevic MP, Lupi KE, Wong A, Gilmore JF, Malloy R (2019). Evaluation of the effect of apixaban on INR in the inpatient population. J Cardiovasc Pharmacol Ther.

[6] Weissenborn K (2019). Hepatic encephalopathy: definition, clinical grading and diagnostic principles. Drugs.

